# Immune Checkpoint Inhibitor-Induced Hypophysitis Presenting as Severe Hyponatremia: A Nephrology Perspective on Avoiding the Syndrome of Inappropriate Antidiuretic Hormone (SIADH) Pitfall

**DOI:** 10.7759/cureus.109633

**Published:** 2026-05-25

**Authors:** Said Al Zein

**Affiliations:** 1 School of Medicine, University of Pittsburgh, Williamsport, USA

**Keywords:** adrenal insufficiency, central hypothyroidism, hyponatremia, hypophysitis, immune checkpoint inhibitor, ipilimumab, irae, nivolumab, pituitary, siadh

## Abstract

A 79-year-old woman with malignant melanoma receiving ipilimumab and nivolumab presented with headache, generalized weakness, nausea, vomiting, and severe euvolemic hyponatremia. Her sodium had declined from 136 mmol/L to 111 mmol/L over one week. Initial urine studies showed hypotonic hyponatremia with inappropriately concentrated urine and elevated urine sodium, mimicking the syndrome of inappropriate antidiuretic hormone secretion (SIADH). Nephrology consultation identified central adrenal insufficiency, with morning cortisol at 1.6 mcg/dL and suppressed adrenocorticotropic hormone (ACTH), along with central hypothyroidism in the setting of immune checkpoint inhibitor therapy.

Glucocorticoid replacement was initiated prior to levothyroxine, and tolvaptan was used as a temporizing aquaretic therapy for persistent severe hyponatremia while hormone replacement was initiated. Sodium normalized, and the patient was transitioned to outpatient endocrine management. This case highlights the importance of evaluating hypophysitis and pituitary failure in patients receiving immune checkpoint inhibitors who present with severe or refractory hyponatremia. Early recognition can prevent delayed glucocorticoid replacement and adrenal crisis.

## Introduction

Immune checkpoint inhibitors (ICIs), including ipilimumab and nivolumab, have transformed the treatment of advanced melanoma but are associated with immune-related adverse events, including endocrine toxicity [1,2]. Hypophysitis is a recognized complication, particularly with cytotoxic T-lymphocyte-associated protein 4 (CTLA-4) inhibitor-based therapy and combination regimens [1,3,4]. It typically presents with nonspecific symptoms such as headache, fatigue, nausea, weakness, and hyponatremia, most often due to central adrenal insufficiency, with or without concomitant central hypothyroidism [2,5]. Reported incidence of hypophysitis ranges from 1-18% depending on regimen, with the highest rates seen in CTLA‑4-based combination therapy [1].

Nephrologists are frequently consulted for hyponatremia in oncology patients, where initial attribution is often made to the syndrome of inappropriate antidiuretic hormone secretion (SIADH), medications, nausea, or malignancy-related causes [5]. However, in patients receiving immune checkpoint inhibitors, SIADH-like urine studies do not exclude pituitary failure [2,5]. This creates a diagnostic pitfall at the point of first specialty assessment, where unrecognized hypophysitis may delay glucocorticoid replacement and increase the risk of adrenal crisis [2,6].

We report a case of immune checkpoint inhibitor-induced hypophysitis diagnosed during nephrology consultation for severe hyponatremia, emphasizing the need to consider pituitary axis dysfunction early in the evaluation of hyponatremia in this patient population.

## Case presentation

A 79-year-old woman with malignant melanoma presented to the emergency department five days after receiving cycle 4 of ipilimumab and nivolumab with headache, generalized weakness, and hypertensive urgency, with systolic blood pressure in the 200s mmHg. Serum sodium at that visit was normal at 136 mmol/L (reference range, 136-145 mmol/L). She was discharged with hydrochlorothiazide added to her antihypertensive regimen.

However, her headache and weakness persisted and worsened over the following week, with the development of nausea and vomiting, leading to hospital admission. On admission, she was afebrile, with blood pressure 157/86 mmHg, heart rate 77 beats/min, respiratory rate 19 breaths/min, and oxygen saturation 100% on room air. She was alert and oriented, not in acute distress, and appeared euvolemic on examination. Cardiopulmonary and abdominal examinations were unremarkable, with no peripheral edema or focal neurologic deficits.

Admission sodium was 111 mmol/L, with serum osmolality 243 mOsm/kg (reference range, 280-300 mOsm/kg), urine osmolality 547 mOsm/kg, and urine sodium 79 mmol/L, showing hypotonic hyponatremia with inappropriately concentrated urine and elevated urine sodium. Creatinine was 1.0 mg/dL, and potassium was 3.3 mmol/L (Table 1). Head computed tomography (CT) and computed tomography angiography (CTA) were negative. Nephrology was consulted, and she was admitted to the intensive care unit.

**Table 1:** Admission laboratory evaluation

Parameter	Value	Units	Reference Range
Serum sodium (baseline)	136	mmol/L	136-145
Serum sodium (admission)	111	mmol/L	136-145
Serum osmolality	243	mOsm/kg	280-300
Urine osmolality	547	mOsm/kg	Not applicable
Urine sodium	79	mmol/L	Not applicable
Creatinine	1.0	mg/dL	0.6-1.2
Potassium	3.3	mmol/L	3.5-5.1
Sodium (hospital day 5)	130	mmol/L	136-145
Sodium (discharge follow-up)	137	mmol/L	136-145

Hydrochlorothiazide was discontinued, and treatment was started with fluid restriction to 1 L/day and salt tablets 1 g four times daily. Sodium showed no meaningful improvement over the next 24 hours. Morning cortisol returned at 1.6 mcg/dL (reference range, 6.9-19.4 mcg/dL), with adrenocorticotropic hormone (ACTH) <5 pg/mL (reference range, 6-50 pg/mL). Thyroid-stimulating hormone (TSH) was 0.262 µIU/mL (reference range, 0.550-4.780 µIU/mL), and free thyroxine (free T4) was low at 0.32 ng/dL. These findings were consistent with central adrenal insufficiency and central hypothyroidism.

Although recent hydrochlorothiazide exposure may have contributed to the rapid decline in serum sodium, the profound central adrenal insufficiency, central hypothyroidism, persistent headache, and recent immune checkpoint inhibitor exposure supported immune checkpoint inhibitor-induced hypophysitis as the primary diagnosis. High-dose methylprednisolone 50 mg every 8 hours was initiated before thyroid hormone replacement. Tolvaptan 7.5 mg once was administered after three days of minimal sodium improvement (112-116 mmol/L) to provide temporary aquaresis while awaiting the effect of glucocorticoid replacement. Sodium rose to 124 mmol/L the next morning and reached 130 mmol/L by hospital day five (Table 2).

**Table 2:** Sodium trend Sodium values are above in mmol/L. Reference range: 136-145

Hospital Day	Time	Sodium (mmol/L)
Day 1	08:17	112
Day 1	12:03	113
Day 1	18:03	112
Day 1	21:17	112
Day 2	00:40	114
Day 2	09:02	113
Day 2	16:53	116
Day 3	03:55	116
Day 3	12:04	117
Day 3	20:02	116
Day 4	04:07	120
Day 4	11:52	125
Day 4	17:56	126
Day 4	23:55	127
Day 5	04:29	128
Day 5	13:43	129
Day 5	20:06	130
Day 6	07:08	130
Day 6	14:19	132
Day 7	06:21	134
2 weeks post-discharge	-	137

Endocrinology confirmed immune checkpoint inhibitor-induced hypophysitis with central adrenal insufficiency and central hypothyroidism. Additional pituitary testing (Table 3) showed inappropriately low follicle-stimulating hormone (FSH) of 7.8 mIU/mL for a postmenopausal woman (reference range, 16.7-113.6 mIU/mL) and luteinizing hormone (LH) of 1.6 mIU/mL (reference range, 10.9-58.6 mIU/mL). Insulin-like growth factor 1 (IGF-1) was 69 ng/mL (reference range, 34-245 ng/mL), and prolactin was normal at 6.3 ng/mL (postmenopausal reference range, 2.74-19.64 ng/mL). Steroids were subsequently tapered, and levothyroxine 75 mcg daily was started after glucocorticoid coverage. The patient stabilized and was discharged on oral hydrocortisone 20 mg in the morning and 10 mg in the early afternoon, levothyroxine, and urea powder. Outpatient follow-up approximately two weeks after discharge showed normalized sodium, and urea was discontinued. Creatinine remained stable at 0.9-1.0 mg/dL throughout hospitalization. Given the absence of visual symptoms or mass‑effect findings, MRI was deferred to outpatient evaluation.

**Table 3:** Endocrine axis evaluation ACTH: adrenocorticotropic hormone; FSH: follicle-stimulating hormone; IGF-1: insulin-like growth factor 1; LH: luteinizing hormone; Prolactin: prolactin; TSH: thyroid-stimulating hormone; Free T4: free thyroxine.

Parameter	Value	Units	Reference Range
Morning cortisol	1.6	µg/dL	6.9–19.4
ACTH	<5	pg/mL	6–50
TSH	0.262	µIU/mL	0.550–4.780
Free T4	0.32	ng/dL	0.8–1.8 (typical)
FSH (postmenopausal)	7.8	mIU/mL	16.7–113.6
LH	1.6	mIU/mL	10.9–58.6
IGF-1	69	ng/mL	34–245
Prolactin	6.3	ng/mL	2.74–19.64

## Discussion

Immune checkpoint inhibitor-induced hypophysitis is a recognized endocrine immune-related adverse event, particularly with CTLA-4 inhibitor-based therapy and combination regimens such as ipilimumab plus nivolumab [1-3]. Although the reported incidence varies by regimen, dose, and surveillance intensity, combination therapy and anti-CTLA-4 exposure carry a higher risk of pituitary toxicity than anti-programmed cell death protein‑1 (anti‑PD‑1) monotherapy or anti-programmed death‑ligand 1 (anti‑PD‑L1) monotherapy [1,2]. Clinical presentation is often nonspecific and may include headache, fatigue, nausea, weakness, and electrolyte abnormalities, making diagnosis difficult in patients with advanced malignancy, recent medication changes, and overlapping treatment-related symptoms [2-4].

This case illustrates a common diagnostic trap in nephrology consultation: severe hypotonic hyponatremia with inappropriately concentrated urine and elevated urine sodium may resemble SIADH, but SIADH‑like urine indices can also occur in adrenal insufficiency or hypothyroidism. Cortisol deficiency results in loss of tonic inhibition of ADH secretion, leading to impaired free water excretion and producing a biochemical picture that can mimic SIADH [3,5]. In this patient, the rapid decline in serum sodium from 136 mmol/L to 111 mmol/L over one week, recent hydrochlorothiazide exposure, nausea, and euvolemic examination initially supported a broad differential including thiazide-associated hyponatremia and SIADH. However, the severity of hyponatremia, persistent headache, limited early response to fluid restriction and salt tablets, and recent immune checkpoint inhibitor exposure prompted pituitary-adrenal axis evaluation. Although recent thiazide exposure can also raise urine sodium and mimic SIADH, the profound central adrenal insufficiency and central hypothyroidism provided a unifying explanation for the patient’s biochemical profile.

The key diagnostic finding was profound central adrenal insufficiency, with morning cortisol at 1.6 mcg/dL and suppressed ACTH. This was accompanied by central hypothyroidism and inappropriately low gonadotropins for a postmenopausal woman, supporting immune checkpoint inhibitor-induced hypophysitis rather than isolated SIADH or thiazide effect. Current guidance recommends hormonal evaluation in suspected ICI-related hypophysitis, including morning cortisol, ACTH, thyroid function testing, gonadotropins, and other pituitary axes when clinically appropriate [2,6]. Dedicated pituitary MRI can support the diagnosis, especially when headache, visual symptoms, or concern for mass effect is present, but normal imaging does not exclude ICI-related pituitary dysfunction [2,3].

Management depends on severity and presence of mass effect, but prompt glucocorticoid replacement is critical when central adrenal insufficiency is identified or strongly suspected. Thyroid hormone should be started only after glucocorticoid coverage because levothyroxine can increase cortisol clearance and precipitate adrenal crisis in an untreated cortisol-deficient patient [3,6]. In this case, high-dose methylprednisolone was initiated before levothyroxine, and tolvaptan was used as a temporizing measure for persistent severe hyponatremia while hormone replacement was initiated. Sodium subsequently improved following glucocorticoid replacement, supporting endocrine-mediated hyponatremia rather than isolated SIADH.

For nephrologists, the practical teaching point is that severe or refractory hyponatremia in patients receiving immune checkpoint inhibitors should prompt early evaluation for adrenal insufficiency and hypophysitis, especially when accompanied by headache, fatigue, nausea, vomiting, or evolving thyroid abnormalities. Recent thiazide exposure or SIADH-like urine indices should not prematurely close the differential. This case emphasizes the nephrology consultation as the diagnostic pivot: recognizing pituitary failure behind SIADH-like laboratory findings allows treatment to address the endocrine driver rather than the sodium abnormality alone, while preventing adrenal crisis and guiding safe thyroid hormone initiation.

## Conclusions

Nephrologists evaluating hyponatremia in patients receiving immune checkpoint inhibitors should maintain a high index of suspicion for hypophysitis and central adrenal insufficiency, particularly when hyponatremia is severe, refractory, or accompanied by headache, fatigue, nausea, vomiting, or evolving thyroid abnormalities. SIADH-like urine studies do not exclude pituitary failure. Early morning cortisol and ACTH testing, followed by timely glucocorticoid replacement before thyroid hormone when central hypothyroidism is suspected, can prevent adrenal crisis and guide appropriate endocrine co-management. This case highlights the role of nephrologists in recognizing endocrine-immune-related adverse events that may initially present as severe hyponatremia.
